# Supplementary material for: Direct evidence of hidden local spin polarization in a centrosymmetric superconductor LaO0.55 F0.45BiS2
Source: Nat Commun. 2017 Dec 4;8:1919. doi: 10.1038/s41467-017-02058-2 (PMC5715082; doi:10.1038/s41467-017-02058-2)
Supplement: Supplementary file 1 — Supplementary Information [file 41467_2017_2058_MOESM1_ESM.pdf]

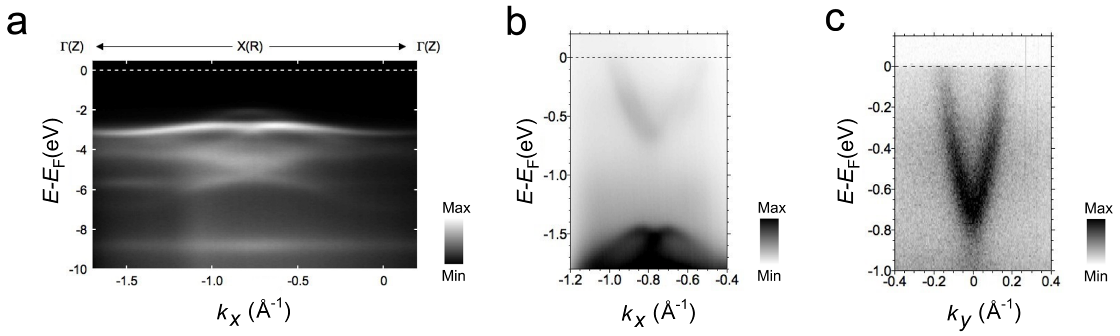

Supplementary Figure 1 | Electronic structure observation of $LaO_{0.55}F_{0.45}BiS_2$ by angle resolved photoemission (ARPES). (a) The raw ARPES data of $LaO_{0.55}F_{0.45}BiS_2$ taken with *hν*=70 eV along Γ(Z)-X(R) line. (b) The raw ARPES data of highest valence band (HVB) and lowest conduction band (LCB) taken with *hν*=18 eV at around X(R) point. (c) The raw ARPES data of lowest conduction band (LCB) along M(A)-X(R)-M(A) line.

Supplementary Note 1. **The raw data of observed band structure by ARPES.**

Since the raw data of angle resolved photoemission spectroscopy (ARPES) was not so clear we showed the band structure obtained by second derivative of energy distribution curves in the main text. However, in order to compare the data with previous paper or future strudies properly we show the row data of Fig. 1d, 1e and 2c in Supplementary Figure 1.

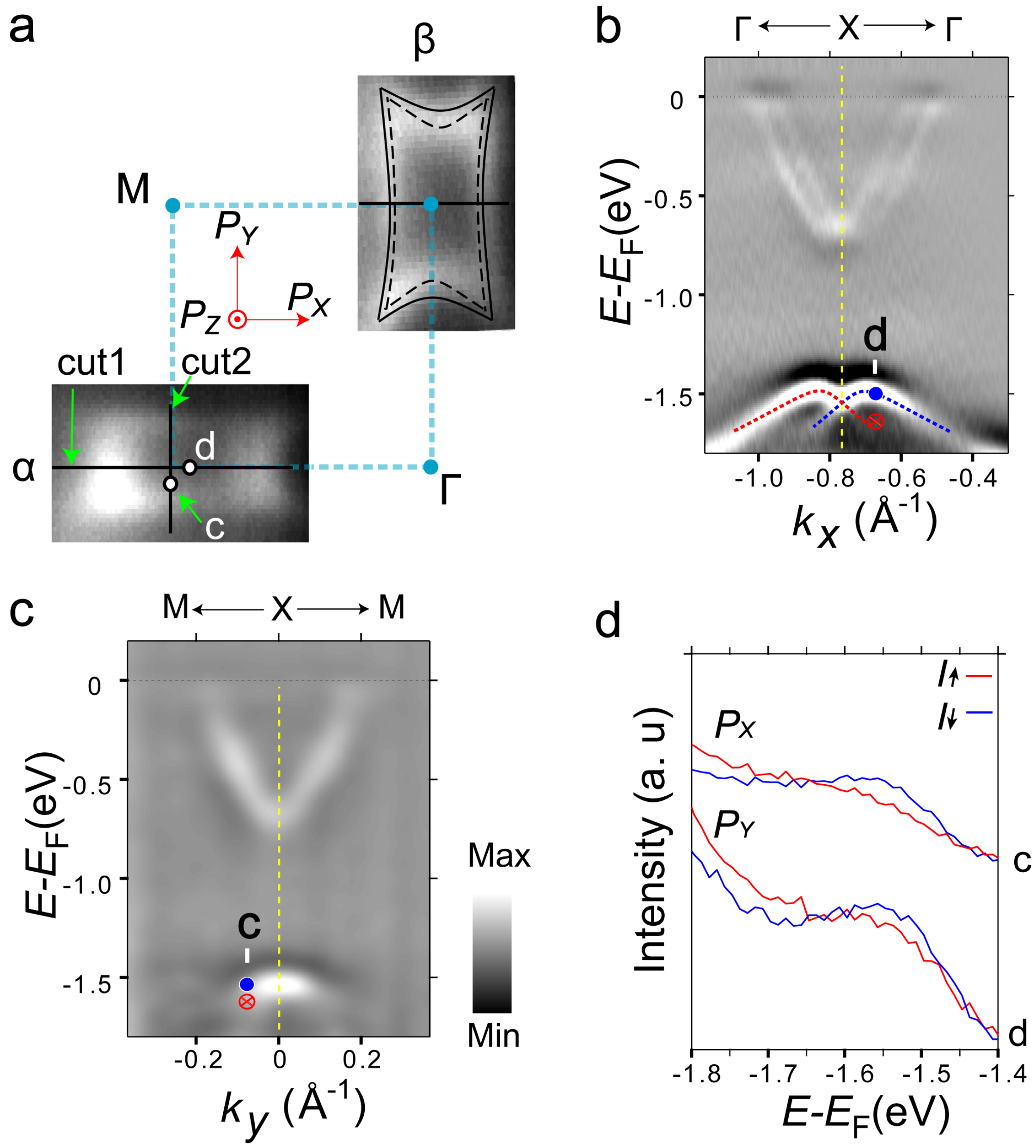

Supplementary Figure 2 | SARPES measurement of highest valence band (HVB). (a) The α and β Fermi surface sheets (FSs) of LCB and the DFT calculation of FSs (black lines). The white dots c and d around X point denote the momentum points where we performed spin measurements in HVB. Coordinate axes (P_X, P_Y, P_Z) denote positive directions of spin vectors. (b) Band dispersion along the cut 1 (Γ-X-Γ line, second derivative). The dashed red and blue lines represent extracted peak positions from the EDCs used for the estimation of Rashba parameter. (c) Band dispersion along the cut 2 (M-X-M line, second derivative). (d) Spin-resolved EDCs of P_X at point c and P_Y at point d for HVB. The red crosses and blue dots in Supplementary Figure 2b and 2c stand for spin-up and down states of spin vectors respectively, corresponding to that of 2d.

Supplementary Note 2. Spin-resolved ARPES results of HVB.

We have investigated the spin-polarization of HVB. Supplementary Figure 2 presents the spin-

resolved EDCs of HVB at positions c and d and the peaks of spectra are also plotted in the Figure. The results indicate clear spin polarizations at the outer branch of splitting HVB both along Γ-X-Γ and M-X-M lines. Opposite spin polarizations are observed in the binding energy range of the inner branch indicating again the local Rashba spin polarization in HVB.

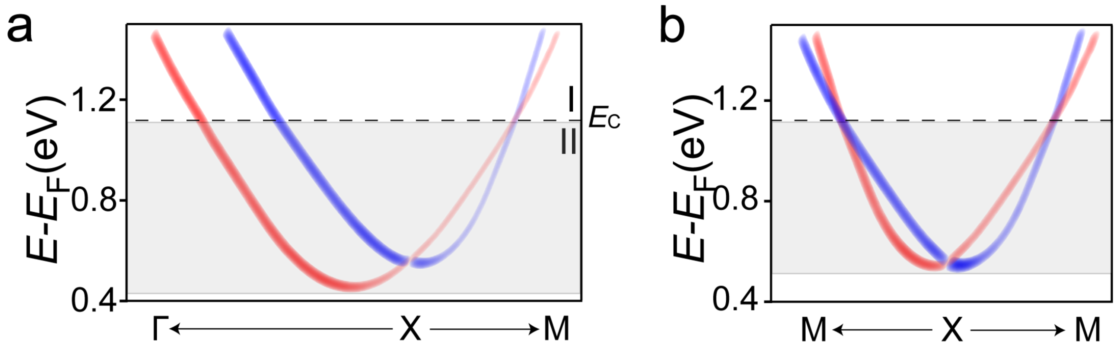

Supplementary Figure 3 | Schematic conduction band (CB) structure around the X point. (a) Schematic conduction band (CB) structure around the X point along the Γ–X–M line in $LaOBiS_2$ parent compounds. A crossing point (E=E_C) along X-M line divides the conduction band into upper (I) and lower (II) regions. (b) The same as (a) but along M–X–M line copied from ref. 1 and 2. Note that the binding energy of our ARPES measurement of CB in $LaO_{0.55}F_{0.45}BiS_2$ crystals differs from the calculated bands with around 1.1 eV because of n-type doping effect. The red and blue colors for energy bands represent spin-up and down states, respectively.

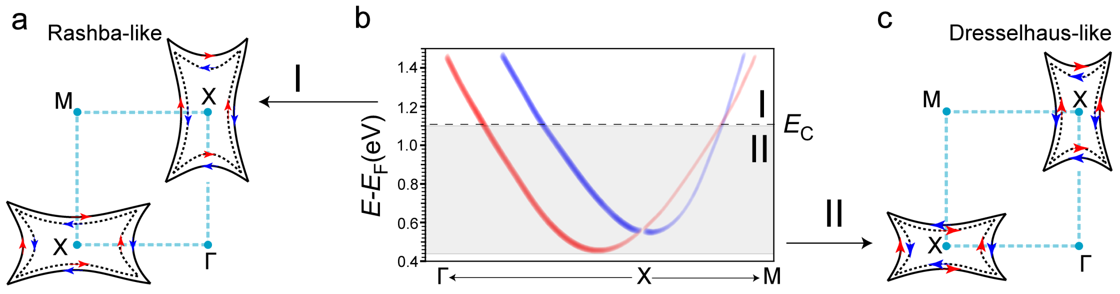

Supplementary Figure 4 | Schematic diagram of spin texture of CB. (a) The Rashba-like spin texture of the rectangle-like shape constant energy contours (CESs) at upper part of CB (region I in Supplementary Figure 3 or 4b). (c) The Dresselhaus-like spin texture of CECs at lower part of CB (region II in Supplementary Figure 3 or 4b) derives from the crossing of spin-polarized CB (E=Ec) along X-M line[1]. The red and blue colors for arrows and energy bands represent spin-up and down states, respectively.

Supplementary Note 3. The conversion of R-2 and D-2 spin textures in conduction band.

Previous theoretical studies[1,2,3] predicted the conduction band (CB) of $LaOBiS_2$ compounds splits into inner and outer branches along Γ–X–M line because of the local spin polarization. Moreover, two branches cross along X–M line leading to a crossing point which divides CB into upper (I) and lower (II) regions shown in Supplementary Figure 3.

The crossing structure of CB has profound effect on spin texture[1]. Namely, the Rashba-like and Dresselhaus-like spin textures of CECs derived from opposite spin polarizations of upper (I) and lower (II) CB along X-M line[1]. The upper band from region I causes the R-2 type helical spin texture while the lower band from region II causes the D-2 type non-helical spin texture, as shown in Supplementary Figure 4. In the main text and the supplementary Note 4 we present spectroscopic evidence of this intriguing spin textures of lowest conduction band (LCB) in n-doped $LaO_{0.55}F_{0.45}BiS_2$ superconductor. The charge transfer from adjacent [LaO] blocks to [BiS_2] by fluorine doping realized the metallization of BiS_2 superconducting bilayers[4], which allows us to observe both conduction band and valence band by (Spin-)ARPES measuring.

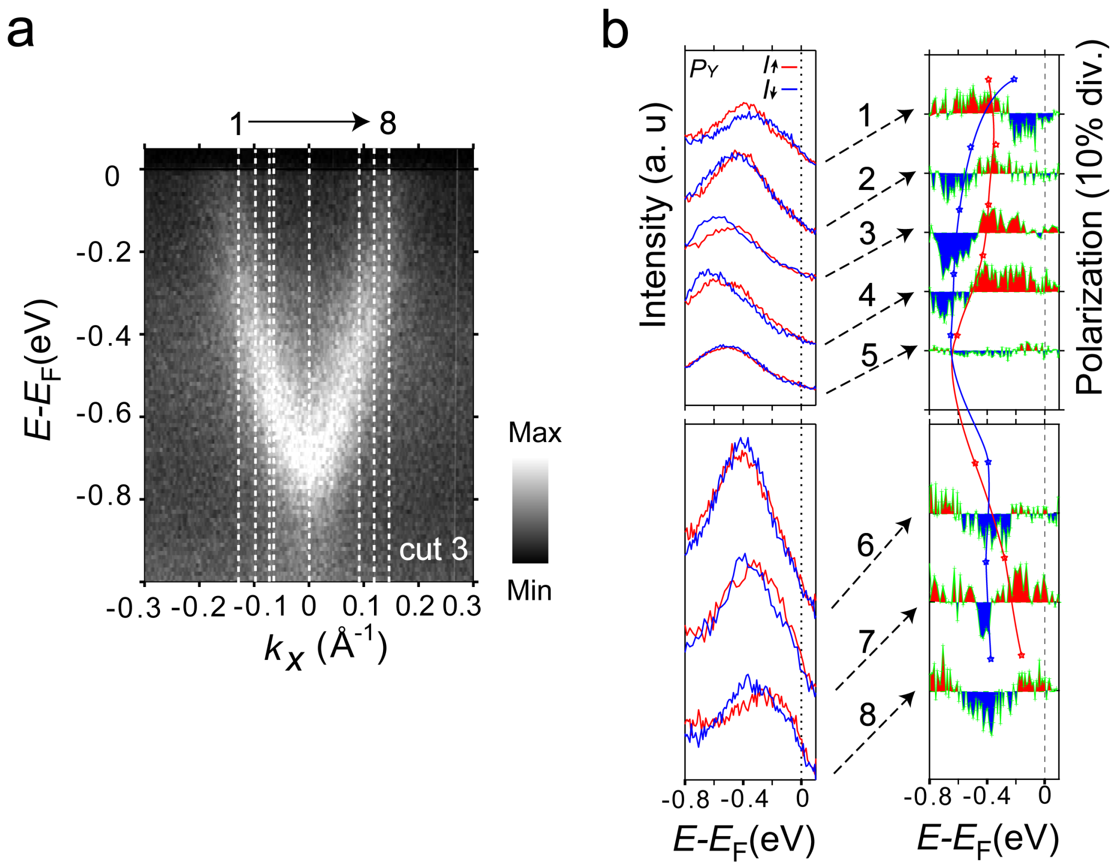

Supplementary Figure 5 | Spin- and angle-resolved photoemission spectroscopy (SARPES) of LCB along cut 3. (a) Band dispersion measured by ARPES ($h\nu$=18 eV) along the cut 3 (M-X-M line). White dashed lines from 1 to 8 mark positions of the energy distribution curves (EDCs). **(b)** Spin-resolved EDCs of P_Y at positions from 1 to 8 shown in (a). The right panel shows the corresponding spin polarizations, the red and blue asterisks in the right panel stand for the fitted peak positions of spin-up and down states of spin vectors, respectively.

Supplementary Note 4. Spin-resolved ARPES results of LCB along X-M line.

Supplementary Figure 5(b) shows the spin-resolved energy distribution curves (EDCs) for E_B = 0-0.8 eV for eight emission angles along M-X-M line indicated by dashed lines in Supplementary Figure 5(a). In Supplementary Figure 5(b), peak shift and polarization change can be observed both at negative k_X positions (1 - 4) and positive k_X positions (6 – 8) with respect to X point (k_X=0). The almost identical spectra in opposite spin channels of P_Y and the cancelled polarization at k_X=0 (position 5) confirm the spin degeneracy of LCB along M-X-M line at X point (Time Reversal Invariant Momenta:TRIM).

With increasing distance from TRIM the spin-resolved EDC spectra from position 4 to 1 demonstrates gradual shift of up- and down-spectra peaks up to position 2 yet the peak positions

reversed at position 1. Note that this crossing position should be very near to position 2 because of the relatively small polarization at position 2.

Moreover, the counter shift behavior has been observed on opposite sides of X point. Namely, the sign of spin polarizations at position 2 is opposite to that of position 6 in the polarization spectra. And we can see again the peak position reversal from position 6 to 7 and 8 in spin-resolved EDCs. The smooth connection of the red and blue asterisks in polarization figure which represent the fitted peak positions of spin-resolved EDCs, schematically illustrates clearly the spin degeneracy at some *k*-point near the positions 2 and 6, as well as reversed spin-up and spin-down states cross the positions. Consequently, the observed spin-resolved EDCs and its polariations indicate clearly the spin polarization reversal around the Dresselhaus-term derived crossing points both at positive and negative k_X with respect to X point. Note that the spin-EDC spectra of cut 2 are taken only at *k*-points at $|\mathbf{k}|>0.1$ Å^{-1} we did not see the spin polarization change from Rashba type to Dresselhaus type (see Figure. 2c and 2e of the main text).

Supplementary References

1. Zhang, X. *et al*. Hidden spin polarization in inversion-symmetric bulk crystals. *Nat. Phys.* **10**, 387-393 (2014).
2. Liu, Q. *et al*. Search and design of nonmagnetic centrosymmetric layered crystals with large local spin polarization. *Phys. Rev. B* **91**, 235204 (2015).
3. Liu, Q., Guo, Y. & Freeman, A. J. Tunable Rashba effect in two-dimensional $LaOBiS_2$ films: Ultrathin candidates for spin field effect transistors. *Nano. Lett*. **13**, 5264–5270 (2013).
4. Shein, I. R., & Ivanovskii, A. L. Electronic band structure and Fermi surface for new layered superconductor $LaO_{0.5}F_{0.5}BiS_2$ in comparison with parent phase $LaOBiS_2$ from first principles. *JETP Lett.* **96**, 769-774 (2013).
